# Interfacial Compatibility on the Crystal Transformation of Isotactic Poly (1-Butene)/Herb Residue Composite

**DOI:** 10.3390/polym13101654

**Published:** 2021-05-19

**Authors:** Bo Wang, Shuangdan Mao, Fuhua Lin, Mi Zhang, Yuying Zhao, Xiuhong Zheng, Hui Wang, Jun Luo

**Affiliations:** 1 School of Chemical and Biological Technology, Taiyuan University of Science and Technology, Taiyuan 030021, China; weaudo@163.com (S.M.); 1991030@tyust.edu.cn (Y.Z.); 1992023@tyust.edu.cn (X.Z.); 2007050@tyust.edu.cn (H.W.); 2 Key Laboratory of Renewable Energy, Guangzhou Institute of Energy Conversion, Chinese Academy of Sciences, Guangzhou 510640, China; kelin0514@163.com; 3 School of Materials Science and Engineering, Taiyuan University of Science and Technology, Taiyuan 030021, China; 4 Shanxi Chemical Research Institute (Co., Ltd.), Taiyuan 030021, China; 18120166132@163.com; 5 Guangzhou Fibre Product Testing and Research Institute, Guangzhou 510220, China

**Keywords:** isotactic polybutylene-1, herb residue, crystal transformation, interfacial compatibility

## Abstract

Isotactic poly (1-butene) (iPB) has excellent properties which are recognized as a green and energy saving product. However, the most stable and valuable crystal form I had a spontaneous transformation that took as long as seven days to complete. As a special solid waste, the herb residue (HR) is rich in cellulose which has great potential to accelerate the crystal transformation of the iPB. However, the polarity of HR results in the interface compatibility with non-polar iPB. In this study, the HR was modified by silane coupling agent (KH570) to obtain KHR and the iPB/HR composite was prepared. The FTIR spectrum was indicated that the organic functional groups of KH570 successfully graft onto the surface of HR and the water contact angle test was indicated that the hydrophilicity of the KHR was greatly decreased. The complete crystal transformation time is 7 days for iPB, 6 days for iPB+5% HR but only 3 days for iPB+5% KHR. The addition of the HR and KHR improve the thermal stability of the composite and this beneficial effect is more obvious for KHR. After annealing for 5 days, the physical properties value include tensile strength, flexural strength, and HDT of iPB+5% HR reach that of pure iPB after annealing for 7 days, but only 3 days for iPB+5% KHR. The TG analysis and SEM photographs give clear evidence that the beneficial effect of KH570 modified HR on improving the interface compatibility with iPB.

## 1. Introduction

Isotactic poly (1-butene) (iPB) has excellent properties of strong long-term circumferential stress bearing capacity, good creep resistance, stress cracking resistance and complete recovery which was widely used in pipe, sheet, film and so on [1]. In particular, the iPB pipe has the advantages of low energy consumption and no scaling which is recognized as a green and energy saving product. As we know, iPB is a polycrystalline polymer that has four kinds of crystal form. The metastable crystal form II formed after molding which has a melting temperature of 110–120 °C. The most stable and valuable crystal form I with a melting temperature of 120–135 °C was spontaneous transformation at room temperature which takes as long as 7 days to complete [2]. Therefore, the long crystal transformation time leads to low production efficiency, slow circulation rate and other problems. Moreover, the shrinkage and density increase during the crystal transformation process leads to the poor dimensional stability of iPB [3]. In recent years, researchers have used the methods of controlling crystallization temperature [4], high-pressure treatment [5], applying tensile stress [6], self-seeding [7], copolymerization with α-olefins [8] to accelerate the crystal transformation rate of iPB. However, the methods are complicated and not suitable for industrial applications. Therefore, blending modification is still a promising way to accelerate the crystal transformation rate. Especially for the high price of iPB, looking for cheap filler for iPB blending modification is the development direction of the iPB modification field. Biomass materials have been widely used in the field of polymer modification due to their low cost, excellent mechanical properties and excellent microstructure which can accelerate the crystallization rate of polymers by heterogeneous nucleation [9,10,11].

Herb medicine is widely used for disease treatment [12]. As a special solid waste, the by-product herb residue (HR) has advantages such as easy collection, low price, good reaction activity, low ash content which is considered as a promising biomass resource [13,14]. The main components of HR are cellulose, hemicellulose and lignin, which are essentially the same as biomass fiber. Therefore, the properties of HR are the same as that of biomass fiber. In previous researches, HR was reused for produce organic fertilizer, feed additives, pyrolysis gasification, papermaking, and biological fermentation for preparation of ethanol, etc [15]. But because of the diversity and complexity of the HR component, there are potential safety problems in the process of reusing HR [16]. Therefore, how to reuse and recycle HR effectively and environmentally friendly is necessary and essential from the viewpoint of industry utilization.

As a kind of biomass fiber, there are a few studies of HR used in polymer blending modification. However, there are lots of hydroxyl groups on the surface of polarity HR which caused the problem of the interface compatibility with polymers [17,18,19]. Feng et al. [20] mixed the HR with polyvinyl alcohol (PVA), and then carried out steam explosion treatment. The hydroxyl group on the molecular chain of PVA can form hydrogen bonds with the hydroxyl group on the surface of HR. The compatibility of polypropylene (PP) and HR was further improved through the bridge effect of PVA. The tensile strength and tensile modulus of PP/HR composite increased by 16.9% and 40.1%, respectively. Yan et al. [21] used steam explosion treatment on HR and prepared HR/polylactic acid (PLA) composite. The results were indicated that the HR in the composite with high lignification degree and less hemicellulose had better mechanical properties. Although the reported methods of modifying the interfacial compatibility of HR and polymer are relatively simple. Actually, the modification methods of heat treatment [22], alkali treatment [23,24], esterification treatment [25,26], coupling agent treatment [27,28] and compatibilizer treatment [29,30,31] were used for improving the interfacial compatibility of biomass fiber and polymer.

In this study, the HR was blending with iPB to prepare iPB/HR composite in order to accelerate the crystal transformation rate of the iPB. The silane coupling agent (KH570) was used to improve the interfacial compatibility. The crystal transformation kinetics was characterized by differential scanning calorimeter (DSC). The interfacial compatibility between iPB and HR was investigated by thermogravimetric analyzer (TGA) and scanning electron microscope (SEM). The Fourier transform infrared spectroscopy (FTIR) and water contact angle were used to determine the effect of KH570 modified HR. The mechanical properties, heat deflection temperature (HDT) of the iPB/HR composites were also investigated in detail.

## 2. Materials and Methods

### 2.1. Materials

The iPB with grade P5050 was purchased from Mitsui Chemical Inc. (Tokyo, Japan). γ-(methacryloxypropyl) trimethoxy silane (KH570) was supplied by Nanjing Xuyang Chemical Co., Ltd. (Nanjing, China). Ethanol was purchased from Sinopharm Group Co., Ltd. (Beijing, China). The crude HR was supplied by Shanxi Hospital of traditional Chinese Medicine (Taiyuan, China). The crude HR was first washed with water and ethanol then dried for 48 h at 100 °C to constant weight. Then the crude HR was crushed in a ball mill for 48 h (DECO-PBM-V-0.4L, Changsha Deco Equipment Co., Ltd., Changsha, China). The HR was obtained after take the crude HR particles less than 200 mesh.

### 2.2. The Modification of HR

The KH570-ethanol solution (10% *w*/*w*) and HR with a mass ratio of 1:50 (*w*/*w*) were mixing in the high-speed mixer (SHR-50, Zhangjiagang Yili Machinery Co., Ltd., Zhangjiagang, China) for 2000 rpm and 20 min. The solid products were dried for 12 h at 80 °C which were named KHR.

### 2.3. Characterization of the KHR

The Fourier transform infrared spectroscopy (FTIR) was performed using the FTIR spectrometer with ATR accessories (Nicolet iS50, Thermo Scientific Inc., Waltham, MA, USA) using 64 scans per sample in order to study the functional group changes of HR after modification.

The HR and KHR sheet was pressed by pressure machine (769YP-10T, Shanghai Xinnuo Instrument Equipment Co., Ltd., Shanghai, China). The water contact angle measuring instrument (DSA25, Kruss, Hamburg, Germany) was used to study the polarity changes of HR after modification.

### 2.4. Preparation of the iPB/HR Composite

The HR and KHR with 5% mass ratios were mixed uniformly with iPB in a high speed mixer (SHR-500A, Zhangjiagang Ruiteyou Plastic Machinery Co., Ltd., Zhangjiagang, China) and the twin-screw extruder (TSH25, Nanjing Chuangbo Machinery Equipment Co., Ltd., Nanjing, China) was used to extrude and pelletize. The temperature for each division of the twin-screw extruder set as 110 °C, 140 °C, 160 °C, 170 °C, 170 °C, and 160 °C, respectively. The dry pellets were molded for standard test specimens by an injection machine (MA1200II, Haitian Plastic Machinery Co., Ltd., Ningbo, China) with an injection pressure of 12 MPa at 150 °C. The composite with 5% HR and 5% KHR was named iPB+5% HR and iPB+5% KHR, respectively.

### 2.5. Characterization of iPB/HR Composite

#### 2.5.1. The Crystal Transformation Rate of the iPB/HR Composite

The crystal transformation rate of the iPB/HR composite was measured by DSC (DSC1, Mettle Toledo Company, Zurich, Switzerland). The samples were first heated to 190 °C at 10 °C/min, maintained for 5 min to eliminating heat history and cooled to 25 °C at 10 °C/min, then annealed at 25 °C for different time (0 day, 1 day, 2 days, 3 days, 4 days, 5 days, 6 days, 7 days,) then heated to 190 °C at 10 °C/min, and the heating curve after annealing was recorded for date analysis.

The melting enthalpies of crystal form I and form II can be calculated by the analysis software of the DSC and the change of crystal form I content (X_I_) with annealing time by the following Equation [32],

(1) XI=ΔHIΔHid,IΔHIΔHid,I+AIIΔHid, II

where ΔH_I_ and ΔH_II_ are the melting enthalpies of crystals in form I and form II, respectively. The ΔH_id,I_ and ΔH_id,II_ are the melting enthalpies of ideal crystals in form I (141 J/g) and form II (62 J/g), respectively [33].

#### 2.5.2. The Interfacial Compatibility of the iPB/HR Composite

The thermogravimetry (TG) analysis was performing on a thermogravimetric analyzer (TGA-1, Mettle Toledo Company, Zurich, Switzerland). The samples were heating up from 30 °C to 600 °C with heating rate was 10 °C/min. The DTG curve is obtained by deriving TG curve data which is used for date analysis.

The SEM analysis was performing on a scanning electron microscopy (JSM-IT200, JEOL, Tokyo, Japan) at an accelerating voltage of 10 kV.

#### 2.5.3. The Physical Properties of the iPB/HR Composite

The physical properties of the iPB/HR composite include mechanical properties and HDT. The samples were annealed at 25 °C for different times (0 day, 1 day, 2 days, 3 days, 4 days, 5 days, 6 days, 7 days,) after molding in order to study the effects of crystal transformation on the physical properties of the composite. For the mechanical properties, the tensile strength and flexural strength of the composite were performed on a universal testing machine (M10, Instron Company, Norwood, MA, USA) according to GB/T 1040-2006 and GB/T 9341-2008. The crosshead speed was set as 20 mm/min. The HDT of the iPB/HR composite was measured by HDT/VICAT equipment (WKW-300B, Changchun Intelligent Instrument Equipment Co., Ltd., Changchun, China) according to GB/T 1634.2-2004. Five samples for each iPB/HR composite were measured and the average values were recorded.

## 3. Result and Discussion

### 3.1. FTIR Spectra of the HR and KHR

**Figure 1:**
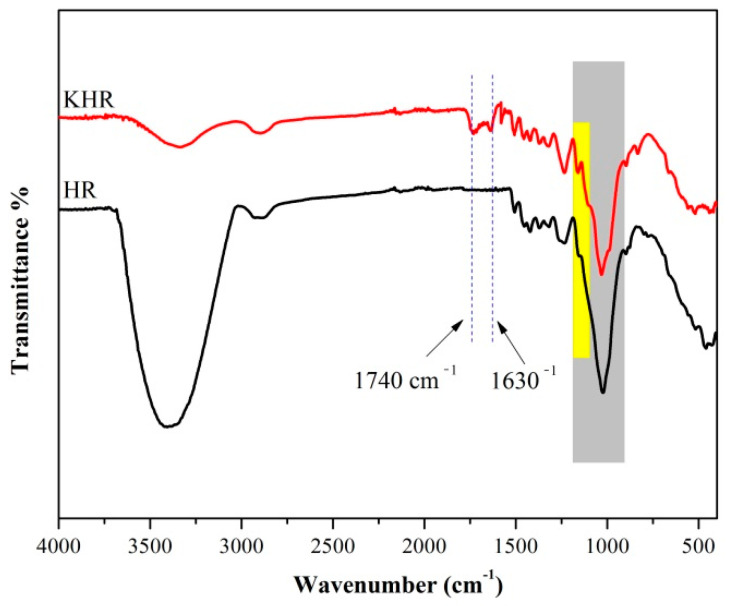
The FTIR spectra of the HR and KHR.

**Figure 2:**
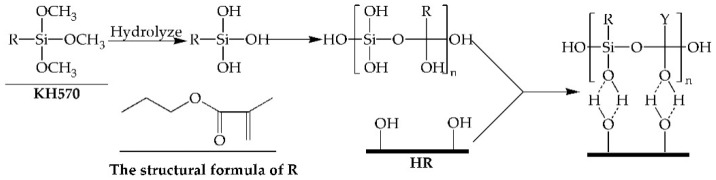
The reaction mechanism of HR and KH570.

The FTIR spectra of the HR and KHR were shown in Figure 1. It can be observed the stretching vibration peak of the C-H and –OH at 2900 cm^−1^ and 3400 cm^−1^, the bending vibration peak of C-H and CH_2_ at 1378 cm^−1^ and 1436 cm^−1^, which relate the characteristic peaks of the HR [34]. After modification, two new peaks attributed to C=O and C=C stretching vibration at 1720 cm^−1^ and 1640 cm^−1^ appeared in the spectra of the KHR, and the peak intensity of –OH decreased significantly [35]. The result indicated that the KH570 has reacted with the –OH of the HR which greatly reduced the hydrophilicity of the HR (Figure 2) [27]. The strong absorption peak of cellulose bond in the range of 1000–1200 cm^−1^ makes it difficult to completely separate the Si-O-Si and Si-O-C peaks [36]. However, due to the overlap of Si-O-Si bonds and C-O stretching of cellulose, the peak value of KHR at 1000–1200 cm^−1^ increased which further proved that the coupling reaction has occurred between KH570 and HR [37].

### 3.2. The Water Contact Angle of the HR and KHR

**Figure 3:**
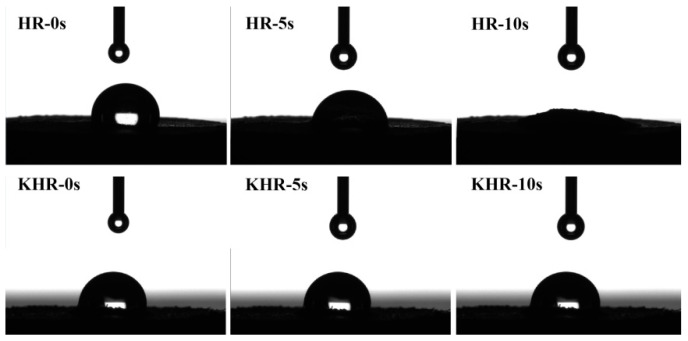
The water contact angle of the HR and KHR.

The water contact angle test is used to determine the polarity of the sample by the different infiltration degree of water droplets on the surface of the sample. In order to extensively study the polarity changes of the HR after being modified by KH570, the water contact angle test was further used for evaluating the hydrophobicity of the HR and the result was shown in Figure 3. It can be seen from the figure that the water droplet was gradually infiltrated into the HR which the water droplet was almost entirely after 10 s and the water droplet is hard to infiltrate into the KHR interior by contraries. This phenomenon indicated that the hydrophilicity of the KHR was greatly decreased due to the organic functional groups of KH570 graft onto the surface of HR which can evidently improve the interface compatibility of the iPB and HR.

### 3.3. The Crystal Transformation Rate of the iPB/HR Composite

**Figure 4:**
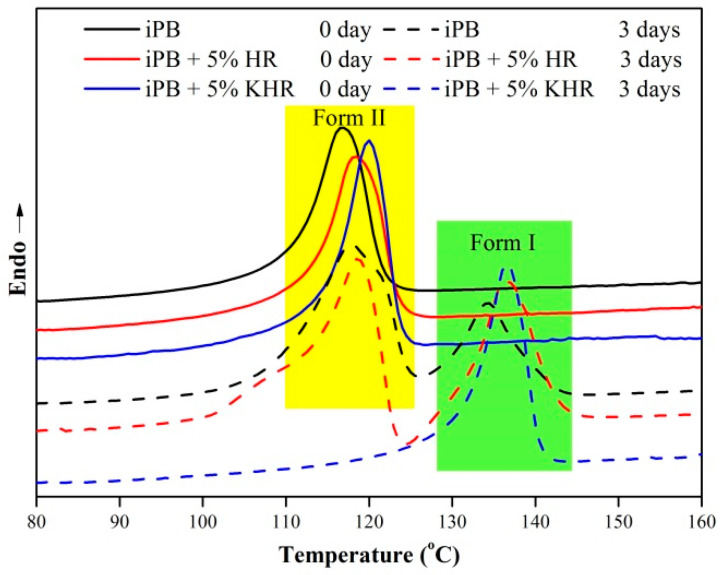
The melting curves of the samples at 25 °C for different annealing time.

The melting curve of the samples after different annealing times at 25 °C is shown in Figure 4. It can be seen from Figure 4 that all samples showed a single melting peak belonging to the form II at the beginning of the molding (0 days). The difference is that the melting temperature of form II (T_mII_) of the iPB+5% HR and iPB+5% KHR are slightly higher than pure iPB. However, the T_mII_ of the iPB+5% KHR is higher than that of the iPB+5% HR. In general, the addition of biomass fiber to the polymer will hinder the movement of molecular chain and increase the thermal stability of the polymer, resulting in an increase in the T_m_ of the polymer. At the same time, the results also prove that the KHR has better compatibility with iPB which resulting in higher Tm_II_ than that of iPB+5% HR. However, the melting peak of form I and form II reappeared in the melting curve of the iPB and iPB+5% HR, and the melting curve of the iPB+5% KHR showed a single melting peak of crystal form I with annealing time for 3 days at 25 °C. Meanwhile, the form I peak area of iPB+5% HR is larger than that of pure iPB. This phenomenon indicates that after 3 days of annealing, some of the crystal form II of iPB, and iPB+5% HR have been transformed into form I, and the conversion rate of iPB+5% HR is faster than that of iPB, while the crystal form of iPB+5% KHR has been completely transformed into form I.

**Figure 5:**
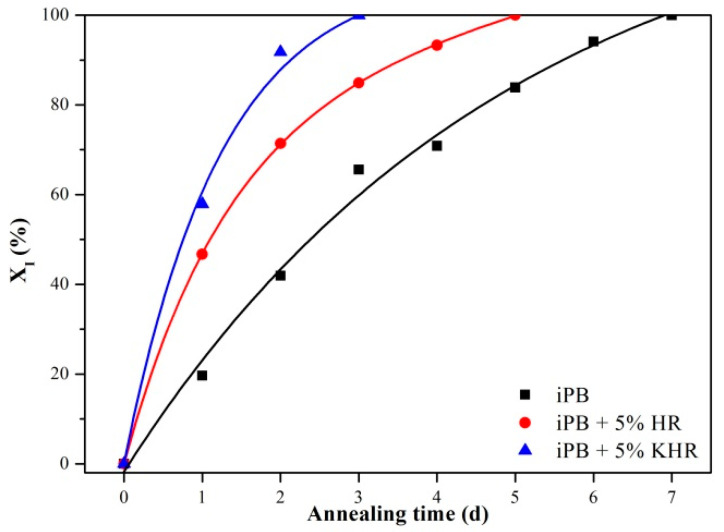
The crystal form transformation kinetics of the samples.

In order to quantitatively compare the accelerating effect of HR and KHR on the crystal transformation of the iPB, the crystal form transformation kinetics of the samples after different annealing times at 25 °C was shown in Figure 5. It can be seen from Figure 5 that the complete crystal transformation time is 7 days for iPB, 6 days for iPB+5% HR but only 3 days for iPB+5% KHR. The result indicates that both the HR and KHR can accelerate the crystal transformation of the iPB. However, the KHR is much better than HR in accelerating the crystal transformation. The reason for this result is that HR plays a good heterogeneous nucleation role in the iPB matrix which accelerates the crystallization rate of form II, thus speeding up the crystal transformation time. Besides, the crystallizing condition and annealing time of the iPB+5% HR and iPB+5% KHR are the same, and the only difference is the interface compatibility with the iPB. This implies that the interface compatibility between HR and iPB is an important factor in accelerating crystal transformation from crystal form II to form I.

### 3.4. The Interfacial Compatibility of the iPB/HR Composite

**Figure 6:**
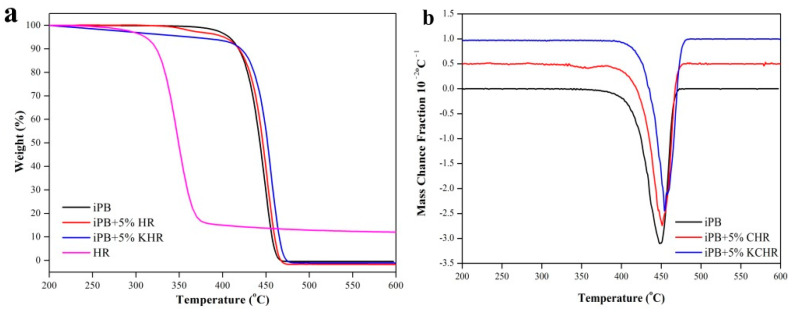
The TG and DTG curve of the iPB/HR composite. ((**a**). TG curve; (**b**). DTG curve).

The TG analysis of the iPB/HR composite was performed in order to study the interfacial compatibility and the thermal stability of the composite which was shown in Figure 6. It can be seen from the figure that the maximum weight loss temperature of the composite increase with the addition of the HR and KHR. This phenomena proves that the addition of the HR and KHR has the benefit to improve the thermal stability of the composite and this beneficial effect is more obvious for KHR. The DTG curves of iPB+5% KHR have only one peak but another small broad peak appeared the DTG curves of the iPB+5% HR. It can be determined that the small broad peak is attributed to the weight loss of the HR by comparing the DTG curve of the HR. The reason for this phenomenon is that the good interfacial adhesion between KHR and iPB produces a strong intermolecular force which shows only one weight loss peak. The poor interfacial compatibility leads to the two weight loss peaks were completely separated and a typical weight loss peak of the HR appeared. It is thus clear that the KH570 modification can greatly improve the interfacial compatibility between HR and iPB.

**Figure 7:**
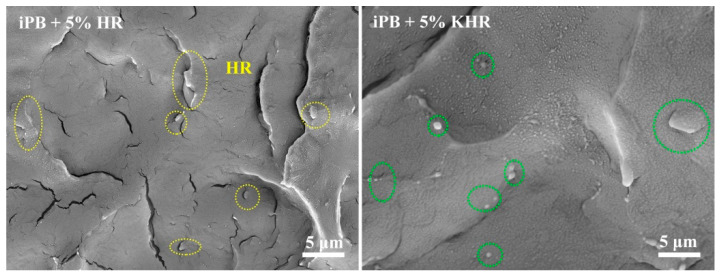
The SEM photographs of the fracture surface of the iPB/HR composite.

The SEM photographs of the iPB/HR composite can be clearly seen as the condition of the compatibility between iPB and HR (Figure 7). The white HR particles and the black iPB matrix can be observed in Figure 7. The difference is the fracture surface of the iPB+5% KHR is flat and smooth and the KHR particle size is small. Contrarily, many crazes can be seen from the fracture surface of the iPB+5% HR and the HR particles size is larger even the phenomenon of particle pull-out occurred. Obviously, the compatibility of HR and iPB is greatly improved after KH570 modification that the composite with more excellent properties can be obtained. The SEM results directly explain the beneficial effect of KH570 modification on improving the interface compatibility between HR and iPB.

### 3.5. The Physical Properties of the iPB/HR Composite

**Figure 8:**
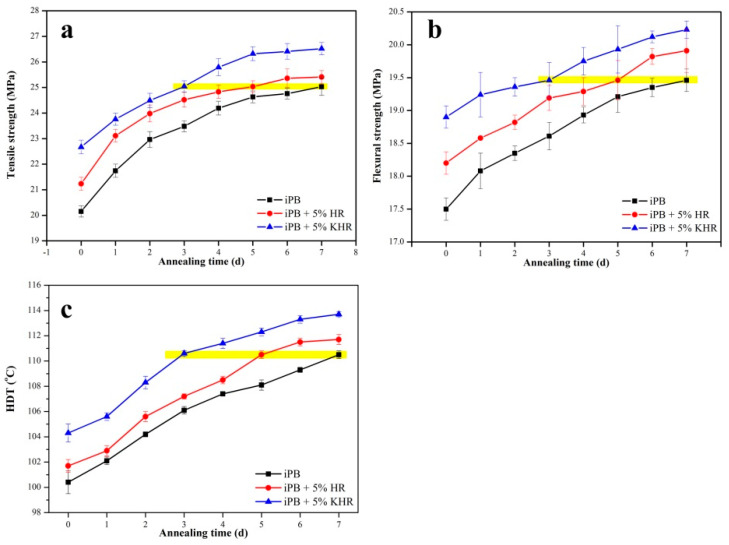
The physical properties of the iPB/HR composite ((**a**). Tensile strength; (**b**). Flexural strength; (**c**). HDT).

The physical properties of polymers are closely related to their crystallization behavior. To this end, the physical properties include tensile strength, flexural strength, and HDT have characterized mechanical and thermal properties of the iPB/HR composite. Several similar phenomena can be seen from the Figure 8 that the physical properties of the composites gradually become better with the extension of annealing time, the physical properties of the iPB+5% HR are better than the pure iPB, and the properties of the iPB+5% KHR are the best.

The reason for this phenomenon is the content of crystal form I in the composites increases with the extension of annealing time, and crystal form I of the iPB has the characteristics of high mechanical strength. Especially, the enhancement effect of the HR further increases the mechanical properties of the iPB. After annealing for 5 days, the physical properties value of iPB+5% HR reach that of pure iPB after annealing for 7 days, and this value was shortened to 3 days in iPB+5% KHR. Besides, it is obvious that the HDT has increased after the crystal form transition is completed after 3 days. The reason for this phenomenon is the HR hinders the movement of molecular chain which cause the increase of the HDT of the composites. From another point of view, this result indicates that better compatibility can not only promote crystal transformation but also improve the physical properties of the composite.

## 4. Conclusions

In this study, the special solid waste HR was modified by silane coupling agent (KH570) and the iPB/HR composite was prepared by the method of melt blending. The new peaks of C=O and C=C appeared in the FTIR spectra and the peak intensity of –OH decreased which indicated that the KH570 successfully graft onto the surface of HR and the water contact angle test indicated that the hydrophilicity of the KHR was greatly decreased. The complete crystal transformation time is 7 days for iPB, 6 days for iPB+5% HR but only 3 days for iPB+5% KHR. The good interfacial adhesion between KHR and iPB produces a strong intermolecular force which shows only one weight loss peak but the poor interfacial compatibility leads to the two weight loss peaks that were completely separated and a typical weight loss peak of the HR appeared. In addition, the fracture surface of the iPB+5% KHR is flat and smooth and the KHR particle size is small and many crazes can be seen from the fracture surface of the iPB+5% HR and the HR particle size is larger even the phenomenon of particle pull-out occurred. The addition of the HR and KHR improve the thermal stability of the composite and this beneficial effect is more obvious for KHR. The physical properties include tensile strength, flexural strength, and HDT of the composites gradually become better with the extension of annealing time, the physical properties of the iPB+5% HR are better than the pure iPB, and the iPB+5% KHR are the best. After annealing for 5 days, the physical properties value of iPB+5% HR reach that of pure iPB after annealing for 7 days, but only 3 days for iPB+5% KHR. The TG analysis and SEM photographs give clear evidence that the beneficial effect of KH570 modified HR on improving the interface compatibility with iPB.

## Data Availability

The data presented in this study are available on request from the corresponding author.
